# Psychological Predictors of Unhealthy Eating Attitudes in Young Adults

**DOI:** 10.3389/fpsyg.2019.00590

**Published:** 2019-03-19

**Authors:** Bernadetta Izydorczyk, Katarzyna Sitnik-Warchulska, Sebastian Lizińczyk, Adrianna Lipiarz

**Affiliations:** ^1^Faculty of Management and Social Communication, Institute of Applied Psychology, Jagiellonian University, Kraków, Poland; ^2^Katowice Faculty of Psychology, SWPS University of Social Sciences and Humanities, Katowice, Poland

**Keywords:** eating attitudes, predictors, young adults, resilience, emotional intelligence, self-esteem, impulsivity

## Abstract

The objective of this study was to determine the predictive role of psychological risk factors for restrained and compulsive eating in young women and men. We examined the relationship between resilience, impulsivity, emotional intelligence and self-esteem, and restrained and compulsive eating. It was assumed that resilience and impulsivity can directly explain unhealthy eating attitudes (restrained and compulsive: both emotional eating and external eating). The study group comprised 211 individuals (105 men and 106 women) aged 20–29, all of whom were living in southern Poland. Measures included the Resilience Measurement Scale (SPP-25), the Eysenck’s Impulsivity Inventory (IVE), the Multidimensional Self-Esteem Inventory (MSEI), the Emotional Intelligence Questionnaire (INTE), and the Polish adaptation of the Dutch Eating Behavior Questionnaire (DEBQ). The statistical analysis showed significant and positive correlations between emotional eating and general self-esteem, impulsivity, and weaker (but still significant) correlations with physical attractiveness. External eating was positively and significantly correlated with impulsivity and self-esteem (including physical attractiveness). Restrained eating was also positively and significantly correlated with general self-esteem. Both types of compulsive eating attitudes (emotional and external eating) were significantly and negatively correlated with resilience. Women showed a significantly higher positive correlation between impulsivity and external eating compared to men. The level of intensity of other measures proved similar across the entire study group regardless of sex. Impulsivity had the strongest and most direct significant influence on both emotional eating and external eating, and a negative effect on emotional intelligence. Resilience proved to have a significant impact on all three examined types of eating attitude (a direct negative effect on emotional eating and external eating, and positive direct effect on restrained eating), self-esteem, and emotional intelligence. An important psychological intervening variable in generating unhealthy eating attitudes proved to be self-esteem among both men and women. Emotional intelligence, which remains correlated with resilience, proved independent, with no effect on unhealthy eating attitudes. These results suggest that preventive treatment and educational programs implemented particularly among adolescents and young adults may support development of their psychological resources.

## Introduction

Contemporary clinical psychology and psychiatry seek knowledge about the risk factors that support growth of unhealthy eating attitudes in young adults. The statistics describing the growing incidence of eating disorders (anorexia, bulimia, binge eating disorder, or obesity among young people) show that there is a high need for extensive scientific research into the identification of psychosocial risk factors vs. protective factors to decrease the incidence of body self-destruction by means of unhealthy eating attitudes (see Smink et al., 2012; Sharan and Sundar Shyam, 2015). Previous literature shows that such unhealthy attitudes often result in deteriorated body and health, with the most common being restrained eating (excessively limited food intake inadequate to maintain good health) and compulsive excessive eating that detracts from good health (Polivy and Herman, 2005; Izydorczyk and Sitnik-Warchulska, 2018). Previous literature also investigates the psycho-social conditions of compulsive and restrained eating attitudes. These studies usually measure selected potential predictors rather than the full range of conditions that lead to restrained or compulsive eating. Research that includes men is also rare. The period of young adulthood associated with taking up different social roles may be affect health behaviors in women and men differently. Massaldjieva et al. (2017) reported that young women declare more disordered eating behavior and risky attitudes compared with young men. These differences, however, are ambiguous.

Many contemporary studies quoted in the literature refer to psychosocial conditions and mechanisms that support various types of eating disorders. The disorders that co-exist with improper eating attitudes most commonly listed in research include various disorders of emotional identity structure with symptoms of emotional lability, anxiety, depression, and obsessive-compulsive disorders (Jacobi et al., 2004; Boyd, 2006; Macht, 2008; Izydorczyk, 2014; Schmidt et al., 2014; Torres et al., 2015; Zipfeld et al., 2015; Dingemans et al., 2017; Lydecker et al., 2018). There are studies that indicate that alexithymia may trigger unhealthy eating attitudes and be related to symptoms of eating disorders, thus strengthening anxiety, depression, and stress(Tchanturia et al., 2012; Torres et al., 2015; Westwood et al., 2017; Brown et al., 2018). There are also studies indicating a lack of evidence to support a significant role for affective disorders in the formation of unhealthy eating attitudes, thus suggesting possible multi-level correlations between improper eating and body attitudes in eating disorders and various affective disorders (Wallis and Hetherington, 2009; Gorwoord et al., 2016; Levinson and Rodebaugh, 2016; Brosof and Levinson, 2017; Wang and Li, 2017). From psychological and medical perspectives, particularly in preventive healthcare and the prevention of eating disorders, seeking the unexplored multi-level influences of various psychosocial conditions on the formation of unhealthy eating attitudes is important for medical and psychological treatment. An analysis of the literature in this area of research reveals that measuring the multi-level impact of psychosocial conditions on restrained and compulsive eating (diversified into external and emotional) is a niche.

There is a need for research to verify the multi-level relationships and correlations between different personal factors and unhealthy eating attitudes in a population of young adults, both women and men. The source material collected over the last years regarding eating disorders (particularly among young women) shows that restrained and compulsive eating, apart from eating disorders, have a significant disorganizing effect on an individual’s psychosocial functioning (Józefik, 2014; Levinson and Rodebaugh, 2016; Brosof and Levinson, 2017; Izydorczyk and Sitnik-Warchulska, 2018). In choosing independent and dependent variables for the original study model, the current authors referred to other researchers’ findings that compulsive and restrained eating can be related to increased levels of depression, anxiety, and low self-esteem (Iniewicz, 2005; Sassaroli et al., 2008; Józefik et al., 2010; Brechan and Kvalem, 2015). Others researchers also indicate correlations between the inability to regulate one’s emotional states and eating patterns (Markey and Vander Wal, 2007; Koifman and Thomas, 2008; Zysberg and Rubanov, 2010; van Strien et al., 2013; Koch and Pallatos, 2014; Ashurst et al., 2018).

The study research objective was to determine the predictive role of psychological risk factors in young adults’ restrained and compulsive eating. Young adulthood usually entails developing self-control, social stability, independence, responsibility, autonomy, abstract thinking skills, the capacity to establish mature interpersonal relations, identity, self-esteem, values, and readiness to perform professional work and determine one’s place in the world. Research in this area is lacking, especially in relation to gender differences. The strength of correlations between resilience, impulsivity, emotional intelligence, and self-esteem, and restrained and compulsive eating was assessed in young women and men. Considering the personal character of resilience and impulsivity, in the model of the original study these two variables were defined as psychological variables that can directly explain unhealthy eating attitudes (restrained and compulsive: emotional eating and external eating). In the model, other variables were also taken into account, although they were assumed to have an indirect effect on the dependent variables. Such location of variables in the research model was determined by the fact that many psychological theories indicate that the nature of emotional intelligence and self-esteem is acquired and forms over a lifetime. According to the Mayer et al. (2000), emotional bonding can be understood as a developmental ability. First, there is the ability to recognize internal emotions based on internal biological reactions and thoughts, and then to perceive and recognize the feelings of others from their facial expressions, behavior or tone of voice. Over a lifetime, the person learns the expression of emotions, develops the skill of empathy and control of emotions, and acquires knowledge about complex emotions and learns to predict emotional states, induce them and use them in various situations (Mayer et al., 2000). In turn, as development theories indicate, self-esteem is the achievement of subsequent developmental stages (see Erikson, 2000). Self-esteem is shaped from an early age, on the basis of contacts with parents, peers, the school environment, or in later stages, the professional environment (Carr, 2009). While during adolescence one can observe a rapid decline in the level of self-esteem (McMullin and Cairney, 2004), along with each subsequent developmental stage, this level will systematically increase (Orth et al., 2010). Hence, it was concluded that these variables (emotional intelligence and self-esteem) will act as intervening variables between the variables of resilience and impulsivity and restrained and compulsive eating Further in the article and discussion on the findings of the original study, the authors apply the name ‘unhealthy attitudes’ interchangeably for restrained and compulsive eating (emotional eating and external eating) as maintaining these attitudes does not promote wellbeing of a study participant.

In the research project two main independent variables were used, namely, resilience and impulsivity. Based on findings of some contemporary research focused on seeking biological grounds for resilience (Russo et al., 2012; Bowes and Jaffee, 2013; Daskalakis et al., 2013) and biological grounds for impulsivity (Moeller et al., 2001; Arce and Santisteban, 2006) it was concluded that both these variables have a status of independent variables. Resilience is variously defined in the literature on the subject. Luthar et al. (2006) indicate that there is confusion around definition, measurement, and interpretation of resilience research. Nowadays resilience is often understood as a process that is related to the functioning of children and youth in crisis situations, and it refers to good adaptation despite overwhelming situations (Masten and Tellegen, 2012). Resilience is also described in the literature as a psychological disposition aimed at protecting an individual against the harmful effect of stressors (Masten, 2001), and as a capacity to overcome failures and obstacles in life (Ogińska-Bulik and Juczyński, 2008; Leipold and Greve, 2009). It is understood as a personality trait, a capacity to cope with stress effectively by means of flexible and creative coping in a difficult situation (Heszen and Sȩk, 2007). Yates et al. (2003) treat resilience as a developmental process, meaning that it occurs in development of every child and is focused on using resources in effective adaptation. Resilience is also treated as a personal feature (see Ogińśka-Bulik and Kobylarczyk, 2015) or internal capacity (Taormina, 2015). In this sense, resilience is not only crucial in a crisis situation, but it is also a feature helpful in dealing with everyday life (Rolin et al., 2018). According to Jackson (2015) average people can use the same principles as people at risk of serious traumatic events to protect their household, or finances from everyday problems. The authors of the present research, referring to other research observations (Izydorczyk and Sitnik-Warchulska, 2018), assumed that every young adult has to struggle with the sociocultural patterns, including those concerning body image. The important relationship between social and cultural impact and resilience is indicated by Masten (2006). Struggling with sociocultural patterns certainly belongs to the stress of everyday life, requiring the mobilization of adaptive sources and abilities such as resilience. Taormina (2015) defined resilience as a multidimensional construct that includes a person’s determination, personal strength to handle difficult situations without giving up (endurance), capacity to be flexible (adaptability), and the physical and mental capacity to recover from adversity (recuperability). Ogińśka-Bulik and Kobylarczyk (2015) also emphasized that resilience consists of many personal features such as elastic adaptation to life’s requirements, persistence in goals, increasing tolerance of negative experiences, the competence of coping with difficulties, openness to new experience, and optimism. Simultaneously, the authors indicate that different ways of understanding resilience complement each other. Resilience should be treated as a dynamic but lasting syndrome, which is determined by many personal and external (social, interpersonal, environmental) factors, and interactions between them (Heszen and Sȩk, 2007; Ogińśka-Bulik and Kobylarczyk, 2015; Taormina, 2015). Such a broad understanding of the concept of resilience was adopted in the present study. Where it is interpreted as a construct (syndrome) manifested as: a capacity to evoke positive emotions (optimism and the ability to mobilize, openness to new experiences and humor) and as the ability to detach from negative experiences (consistency and determination in action, capacity to cope with negative emotions and to tolerate failures) see Ogińska-Bulik and Juczyński, 2008; Ogińśka-Bulik and Kobylarczyk, 2015).

In turn, the variable of impulsivity was defined as a relatively stable tendency of an individual to react in a rapid and unplanned manner in response to an internal impulse or external stimuli (Moeller et al., 2001). In the literature, impulsivity is described most often as a multidimensional construct (a personality trait, a symptom) of a tendency to take excessive risk, unplanned and rapid actions that are ill-considered and inadequate in a given situation, oftentimes related to an inability to postpone gratification (Moeller et al., 2001; Arce and Santisteban, 2006). Increased impulsivity levels manifest most commonly in addicted individuals with suicidal tendencies, aggressive behavior (Jakubczyk and Wojnar, 2009) and people suffering particularly from bulimia (Grzesiak et al., 2008; Manwaring et al., 2011; Forney et al., 2014; Lavender and Mitchell, 2015), as well as those engaging in binge eating (Striegel-Moore and Franko, 2003; Izydorczyk, 2011, 2013; Annagur et al., 2015; Pearson et al., 2015).

In the original study presented in this paper self-esteem and emotional intelligence were also taken into account in the research model. The levels of both self-esteem and emotional intelligence change with age (Mayer et al., 2000), thus, they can be subject to environmental influences. Self-esteem and emotional intelligence are related to cognitive structure development in personality and indicate correlations between thought processes and emotions (Petrides et al., 2004).

Self-esteem is defined in the original research model in accordance with the literature as a formed general way of perceiving oneself. Rosenberg (Łaguna et al., 2007) considers self-esteem as an attitude pertaining to the self that can be either positive or negative. In this sense, self-esteem is related to an external personal perception of oneself and one’s own capabilities. In adolescence self-esteem can be observed to fall rapidly (McMullin and Cairney, 2004), but with every subsequent developmental stage the level increases systematically (Orth and Robins, 2010). As mentioned above, although self-esteem can be altered by key life events, in young adulthood it tends to stabilize and individuals begin to consistently assess themselves in a certain way. Good self-esteem usually motivates one to engage in health-promoting behavior and facilitates psychological wellbeing that allows one to maintain good health (Ogińska-Bulik, 2010; Gruszczyńska et al., 2015). Inadequate self-esteem may result in individuals assessing their health condition in an excessively optimistic way, which can contribute to high risk behavior (Baumeister et al., 2003). Individuals with high self-esteem accept and like themselves, and show higher levels of adaptation, lower levels of neuroticism (Schmitt and Allik, 2005), and higher satisfaction with life (Furnham and Cheng, 2000).

The second intervening variable that explains unhealthy eating attitudes in young adults was identified as emotional intelligence, defined as a trait, competence, or capacity to identify and name feelings expressed verbally and non-verbally, and to control one’s own feelings, i.e., to consciously suppress them or completely focus on them (Mayer et al., 2004). Emotional intelligence is the capacity to draw information from emotional states to improve thought processes, creativity, motivation level or attention flexibility. Emotional intelligence is considered an integral part of social intelligence, since it both affects individuals’ psychological processes and manifests in their interpersonal interactions, while substantially effecting the quality of relations (Goleman, 1997; Mayer et al., 2004; Austin and Saklofske, 2005).

The dependent variables employed in the original study model of the original study are three dominant patterns of unhealthy eating attitudes: restrained and compulsive ones (external and emotional eating). Compulsive behaviors consist in binge eating inadequate to medical recommendations, engaging often in emotional eating. In turn, restrained eating consists in excessive limitation (either qualitative or quantitative) of food intake in one’s daily diet that is inadequate to health requirements. External eating determined as part of compulsive attitudes is defined as behavior consisting in excessive eating triggered by the smell or look of food, as well as by the accessibility to places where one can buy food quickly and effortlessly. External stimuli can be seen by an individual attractive enough to trigger a need to eat even without feeling the physiological sensation of hunger. External eating cause weight gain, which in turn leads one to a decision to limit food intake. In turn, emotional eating is a behavior that consist in compulsive eating of food in excessive amounts to release inner tension caused by various emotions such as boredom, anger, irritation, sullenness, anxiety, fear, disappointment, grief or guilt (Macht, 2008; Allison et al., 2009; Herman and Polivy, 2010; Ogińska-Bulik, 2010). Emotional eating attitudes may constitute also an atypical response to stress. Many studies show that individuals suffering from anorexia who thus manifest excessively restrained eating show a consolidated general low level of emotional functioning (Hambrook et al., 2011; Teresawa et al., 2013; Schmidt et al., 2014; Lang et al., 2015; Oldershaw et al., 2015; Racine and Wildes, 2015). In turn, studies on people with bulimia also show these individuals to manifest disordered emotional structure of personality and strongly consolidated emotional dysregulation with considerable difficulties in describing the emotions they experience, which has a significant effect on unhealthy eating attitudes (Pascual et al., 2011; Brockmeyer et al., 2012; Svaldi et al., 2012; Danner et al., 2014).

Two research questions were put forward:

(1) Do resilience, impulsivity, self-esteem, and emotional intelligence explain restrained and compulsive (emotional and external) eating in young women and men, and if so, to what extent?
(2) Do female and male participants differ in regard to their level of resilience, impulsivity, self-esteem, or emotional intelligence, or their restrained and compulsive eating, and if so, in what way?

## Materials and Methods

### Participants

The study was conducted in 2016 – 2017 and used purposive sampling in a population of young adult men and women who were full-time students attending several universities in southern Poland. Some students in the sample also worked part-time.

Three-hundred 20- to 30-year-old early to young adults were recruited; 89 were excluded from the analysis because of incomplete survey forms, leaving 211 participants (105 men and 106 women) ranging in age from 20 to 29.

The inclusion criteria were: age between 20 and 30 years, no documented eating disorders that required treatment (anorexia, bulimia, binge eating disorder) or other mental disorders. The exclusion criteria were: age below 20 or above 30 years, any documented episode of treatment for eating disorders (anorexia, bulimia, binge eating disorder), obsessive-compulsive disorder, depressive episodes, specific phobias, dysmorphophobia, borderline personality disorder, or psychoactive substance misuse. The exclusion criteria were based on previous psychopathology of eating disorders research that reported an association between other types of mental disorders and improper eating attitudes (Grant et al., 2002; Jacobi et al., 2004; Czepczor and Brytek-Matera, 2017). Individuals who withdrew from the study or failed to complete all questionnaires were excluded from the analyses.

### Compliance With Ethical Standards

This study was conducted in accordance with the recommendations from the Research Ethics Committee of the Institute of Applied Psychology, Jagiellonian University in Krakow and was determined to conform with the 1964 Helsinki declaration and its later amendments or comparable ethical standards. The participants received detailed information on the objective, course, and conditions for participating in the study, and were informed that their participation was voluntary and their data would be kept confidential. All participants provided written informed consent to participate.

The protocol was also approved by the Research Ethics Committee of the Institute of Applied Psychology, Jagiellonian University, Krakow.

### Instruments

The first independent variable, resilience, was measured using the Resilience Measurement Scale (SPP-25) developed by Ogińska-Bulik and Juczyński (2008) A high reliability and accuracy coefficient was obtained in this study (Cronbach’s α = 0.89). The scale contains 25 items scored on a five-point Likert scale from 0 (*strongly disagree*) to 4 (*strongly agree*). The maximum total score is 100, minimum is 0. Scores ranging from 0 to 65 indicate low mental resilience; 66–77 indicate average mental resilience; 78–100 indicate high mental resilience.

The second independent variable, impulsivity, was measured using the Polish version of Eysenck’s Impulsivity Inventory (IVE) developed by Jaworowska (2011). Only the impulsivity scale, with Cronbach’s alpha of 0.70, was used in this study. It contains 19 items with two possible responses, *agree* (1 point) or *disagree* (0 points). The maximum total score is 19, minimum is 0. Low scores (0–4) indicate low impulsivity; 5–15 indicate average impulsivity; high scores (14–19) indicate high impulsivity (Jaworowska, 2011).

The intervening variable, self-esteem, was measured using the Multidimensional Self-Esteem Inventory (MSEI) developed by O’Brien and Epstein (Fecenec, 2008). Based on the results of the original study, three MSEI subscales were employed: global self-esteem, physical attractiveness, and vitality Items use a five-point Likert scale, ranging from 1 (*strongly disagree*) to 5 (*strongly agree*). Additional items require respondents to estimate the frequency of specific situations, feelings, or thoughts occurring in their life, ranging from 1 (*almost never*) to 5 (*very often*). Scores were summed and converted to stens. The global self-esteem subscale contains eight items, with a minimum total score of eight and a maximum of 40. Physical attractiveness and vitality subscales allowed us to include a measure of body self-esteem, which is a motivating factor for changing eating attitudes in response to an unaccepted body image (Izydorczyk and Sitnik-Warchulska, 2018). The physical attractiveness and vitality subscales contain 10 items each, with a maximum score of 50. Each MSEI subscale showed sufficiently high reliability in this study, with Cronbach’s alphas of 0.90 for general self-esteem; 0.88 for physical attractiveness; and 0.90 for vitality.

Emotional intelligence was measured using the Emotional Intelligence Questionnaire (INTE) developed by Schutte et al. (1998) adapted to Polish conditions by Ciechanowicz et al. (2000). It assess emotional intelligence, including the capacity to perceive, evaluate, express, and regulate emotions, and to use them to improve action and thought processes. The INTE contains 33 self-assessed items with five-point Likert scales from 1 (*strongly disagree*) to 5 (*strongly agree*). The total score (33 minimum, 165 maximum) is then converted to stens. A raw score up to 115 for women and 113 for men indicates low emotional intelligence; 116–138 for women and 114–135 for men indicates average emotional intelligence; 139–165 for women and 136–165 for men indicates high emotional intelligence. Cronbach’s alpha in this study was 0.87 (Jaworowska and Matczak, 2008).

There is no standardized measure for restrained and compulsive eating in the Polish psychological literature or diagnostics; therefore the dependent variable was measured with the Dutch Eating Behavior Questionnaire (DEBQ), initially adapted for use Polish populations by van Strien et al. (1986). The English version of the DEBQ contains 33 items in three subscales that measure eating attitudes, restrained eating, external eating, and emotional eating. Each DEBQ subscale has high statistical reliability. The Polish adaptation was developed through a pilot study on a random sample of 199 healthy people that excluded any individuals with a history of clinically diagnosed mental disorders, including anorexia, bulimia, binge eating disorder, and psychotic and/or somatic disorders with concomitant symptoms of appetite disorders. The 33 items of DEBQ were initially translated by English translators into Polish and then reverse-translated. A five-point Likert scale, from 1 (*never*) to 5 (*very often*), measured responses to 33 items describing the frequency of restrained and/or compulsive eating behaviors, where higher scores indicated higher levels of the trail (i.e., restrained eating, or compulsive eating). Statistical factors for all items in the DEBQ were verified and estimated by means of confirmatory factor analysis and the identified factors were subject to a varimax rotation with Kaiser normalization. In the first stage of the analysis the KMO and Bartlett’s test of sphericity were used to determine sampling adequacy. The results showed the sampling to be good [KMO = 0.88; Bertlett’s test of sphericity χ^2^(528) = 3924.06; *p* < 0.001], confirming the soundness of the factor analysis. Next, an exploratory factor analysis was performed with a varimax orthogonal rotation. The results did not confirm the three-factor structure of DEBQ assumed by the authors. To verify whether it is possible to match the obtained results of the pilot study with the three-factor model of the original DEBQ, a confirmatory factor analysis with an orthogonal varimax rotation with the pre-set number of three factors was performed as the next step. The results distinguished three factors that explained 53.5% of the variance as shown in Table 1.

**Table 1:** Results of the confirmatory factor analysis with pre-set number of three factors for DEBQ.

DEBQ factors	The sum of squared loadings after determination			The sum of squared loadings after varimax rotation		
	Total	% variance	% accumulated	Total	% variance	% accumulated
1	8.09	24.53	24.53	6.96	21.08	21.08
2	6.63	20.09	44.61	6.26	18.97	40.05
3	2.95	8.94	53.55	4.46	13.50	53.55

*1, restrained eating; 2, external eating; 3, emotional eating.*

As shown in Table 1, specific positions in DEBQ were successfully ascribed to three main factors, prompting a decision to maintain the three-factor DEBQ structure. The determined sub-scales demonstrated high reliability, with Cronbach’s alpha over 0.90. Three factors identified by factor analysis allowed for three corresponding scales to be distinguished: scale 1 (10 items) was named ‘restrained eating’ (Cronbach’s α = 0.95), scale 2 (10 items) was named ‘external eating’ (Cronbach’s α = 0.90), and scale 3 (13 items) was named ‘emotional eating’ (Cronbach’s α = 0.94). The developed version of the eating attitude questionnaire was then applied in the original studies to measure restrained and compulsive attitudes (external and emotional eating) in a population of 211 young adults.

### Statistical Methods

Data were analyzed using STATISTICA 8 and IBM SPSS Statistics, version 19.0. Descriptive statistics included frequencies and percentages for categorical variables and means with standard deviations for continuous variables. The Kolmogorov–Smirnov test was used to verify normal distributions. *p* < 0.05 level of significance was used for all analyses. In the third stage of statistical analyses, the strength of correlation between all the variables was measured using Pearson’s *r*. The final element of statistical analyses included path analysis to confirm concordance between the theoretical model (research model) and the collected data.

**FIGURE 1:**
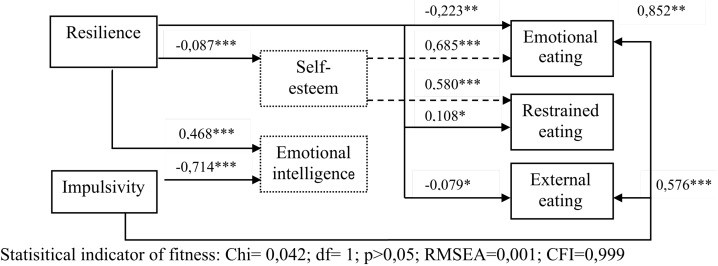
Model of paths of factors explaining unhealthy eating attitudes.

To verify assumptions about the influence of psychological variables on food-related behaviors, structural equation models with path diagrams were used. Using linear equations, causal models were tested to determine to what extent the data agreed with the theoretical causality model. Structural equation modeling was conducted on the full sample without stratification by groups (e.g., by gender) to maintain adequate statistical power. All research variables were introduced into the model. On the basis of the numerical values of individual model parameter estimators, it can be determined whether indirect or direct influences of psychological variables on food-related behaviors exist. Intercorrelations between the dependent variables and the paths that proved to be irrelevant, were omitted. The final image of the fitted model is described in the “Results” section (Figure 1).

## Results

The aim of the analysis was to identify psychological predictors of unhealthy (restrained and compulsive) eating attitudes in the sample of young women and men.

The general characteristics of the study group (*n* = 211 are presented in Table 2.

**Table 2:** Basic descriptive characteristics of the examined quantitative variables in the study group (*n* = 211).

Variables	*M*	Me	Min	Max	*SD*
Emotional intelligence	118.08	120.00	60.00	145.00	12.32
Impulsivity	6.91	6.00	1.00	15.00	3.07
Resilience	89.95	92.00	42.00	123.00	14.44
Self-esteem (general and body self-esteem)	45.80	46.00	32.00	57.00	4.88
Emotional eating	34.21	34.00	13.00	65.00	13.82
Restrained eating	26.36	26.00	10.00	50.00	9.61
External eating	32.50	33.00	14.00	48.00	7.02

Analysis of the means for specific measure identified resilience as the strongest independent variable Self-esteem (including both general and body self-esteem) was below average. Impulsivity and emotional intelligence fell within the range of average results. These results suggest that participants manifested high levels of mental resilience average emotional literacy and impulsivity, and below average general and body self-esteem.

As for the dependent variable describing unhealthy attitudes, results showed restrained eating to be most strongly manifested in the study sample (slightly above average). External and emotional eating were average. Interestingly, the mean results indicating low self-esteem were associated with above-average levels of restrained eating. This may suggest a significant socio-cultural influence on excessive pursuit of thinness among young adults, but this possibility was not tested in our analysis.

In the subsequent stage of the statistical procedure, we measured the strength of correlations among all the variables. Results are presented in Table 3.

**Table 3:** Comparison of Pearson’s *r* values in the examined population of young adults (*n* = 211).

	Emotional intelligence	Impulsivity (IVE)	Resilience (SPP25)	General self- esteem (MSEI)	Physical attractiveness (MSEI)	Vitality (MSEI)	Emotional eating	Restrained eating
Impulsivity (IVE)	−0.268^∗∗∗^							
Resilience (SPP 25)	0.578^∗∗∗^	−0.166^∗∗^						
General self- esteem (MSEI)	−0.160^∗∗∗^	0.127	−0.271^∗∗∗^					
Physical attractiveness (MSEI)	0.039	0.130	0.103	0.372^∗∗∗^				
Vitality (MSEI)	0.137^∗^	−0.014	0.221^∗∗∗^	0.423^∗∗∗^	0.232^∗∗∗^			
Emotional eating (DEBQ)	0.133	0.234^∗∗∗^	−0.277^∗∗∗^	0.314^∗∗∗^	0.170^∗∗^	−0.090		
Restrained eating (DEBQ)	−0.008	0.064	0.050	0.263^∗∗∗^	0.130	0.085	0.176^∗∗^	
External eating (DEBQ)	−0.045	0.258^∗∗∗^	−0.160^∗^	0.181^∗∗^	0.207^∗∗^	−0.093	0.575^∗∗∗^	0.031

*^∗^p < 0.05; ^∗∗^p < 0.01;^∗∗∗^p < 0.001. DEBQ, Dutch Eating Behavior Questionnaire; INTE, Emotional Intelligence Questionnaire; IVE, Impulsiveness-Venturesomeness-Empathy questionnaire; SPP 25, Resilience Measurement Scale; MSEI, Multidimensional Self-Esteem Inventory for general self-esteem, physical attractiveness, vitality.*

A significant correlation was found between emotional intelligence and resilience, with weaker but still significant correlations between impulsivity, general self-esteem, and vitality. Emotional intelligence and vitality were not significantly correlated with any of the unhealthy eating attitudes we tested. Higher emotional intelligence was associated with weaker impulsivity and weaker general self-esteem, but higher vitality.

Resilience significantly positively correlated with emotional intelligence, but negatively correlated with general self-esteem and impulsivity. Impulsivity also showed a significant negative correlation with resilience. Moreover, resilience showed a moderate positive correlation with vitality.

In terms of self-assessment, all of the component variables proved to be positively correlated with each other. General self-esteem was correlated with both body self-esteem and vitality.

There was a significant (but not very strong) negative correlation between emotional eating and resilience, a significant moderate positive correlation between emotional eating and general self-esteem, and a weaker correlation with physical attractiveness. Higher resilience was associated with weaker emotional eating and higher self-esteem was associated with higher emotional eating. Emotional eating was also correlated with impulsivity, but this correlation was not very strong. Emotional eating showed a strong positive correlation with external eating attitude and a significant but weak correlation with restrained eating attitude. External eating was positively correlated with impulsivity levels, general self-esteem and physical attractiveness (significant correlation but weak), but negatively correlated with resilience. Hence, lower levels of resilience are associated with stronger excessive eating triggered by food smell or appearance, or effortless accessibility to food. Restrained eating was significantly correlated only with general self-esteem. Stronger global elf-esteem (but not body image), is associated with higher restrained eating levels.

### Descriptive Characteristics of Variables in the Original Research Model (Comparative Analysis for Female and Male Participants)

The next step of the analysis involved verifying sex-based differences. We used Kolmogorov–Smirnov tests to confirm normal distributions, then Student’s *t* tests to identify group differences. Detailed results are presented in Table 4.

**Table 4:** Comparative analysis of women and men in terms of mean values of the research variables.

	Women (*n* = 106)		Men (*n* = 105)			Cohen’s *d*
Research variables	*M*	*SD*	*M*	*SD*	*t*	
Resilience	90.80	14.39	89.08	14.51	0.871	0.12
Impulsivity	6.82	2.80	7.00	3.32	−0.421	0.11
Emotional intelligence	119.88	10.02	116.26	14.11	2.16^∗^	0.33
General self-esteem	45.98	4.80	45.61	4.98	0.554	0.13
Emotional eating	33.78	13.94	34.65	13.75	−0.46	0.06
External eating	31.41	7.44	33.62	6.40	−2.32^∗^	0.30
Restrained eating	25.71	9.71	27.03	9.49	−0.99	0.13

*M, mean; SD, standard deviation; t, Student’s t test result; ^∗^p < 0.05.*

Results showed that women scored higher on average in emotional intelligence compared to men, whereas external eating was stronger in men. However, the indicated variables fell within the range of mean average results, both in the group of women and the group of men. There were no other significant differences by sex suggesting that sex does not influence unhealthy eating attitudes in any significant way.

Moreover, correlations between the subscales of the Polish adaptation of DEBQ with other questionnaires used in the authors’ study were examined separately in women (Table 5) and men (Table 6).

**Table 5:** Comparison of Pearson’s *r* values in the examined population of young women regarding subscales of the DEBQ with the INTE, IVE, SPP 25 MSEI.

	Emotional eating (DEBQ)	Restrained eating (DEBQ)	External eating (DEBQ)
Emotional intelligence (INTE)	−0.221^∗^	−0.031	−0.176
Impulsivity (IVE)	0.208^∗^	0.142	0.369^∗∗∗^
Resilience (SPP 25)	−0.237^∗∗^	0.001	−0.191^∗^
General self- esteem (MSEI)	0.264^∗∗^	0.256^∗∗^	0.158
Physical attractiveness (MSEI)	0.120	0.073	0.225^∗^
Vitality (MSEI)	−0.062	−0.016	−0.156

*^∗^p < 0.05; ^∗∗^p < 0.01; ^∗∗∗^p < 0.001. DEBQ, Dutch Eating Behavior Questionnaire; INTE, Emotional Intelligence Questionnaire; IVE, Impulsiveness-Venturesomeness-Empathy Questionnaire; SPP 25, Resilience Measurement Scale; MSEI, Multidimensional Self-Esteem Inventory for general self-esteem, physical attractiveness, vitality.*

**Table 6:** Comparison of Pearson’s *r* values in the examined population of young men regarding subscales of the DEBQ with the INTE, IVE, SPP 25 MSEI.

	Emotional eating (DEBQ)	Restrained eating (DEBQ)	External eating (DEBQ)
Emotional intelligence (INTE)	−0.067	0.025	0.104
Impulsivity (IVE)	0.257^∗∗^	−0.007	0.150
Resilience (SPP 25)	−0.316^∗∗∗^	0.109	−0.108
General self- esteem.(MSEI)	0.368^∗∗∗^	0.276^∗∗^	0.229^∗^
Physical attractiveness (MSEI)	0.224^∗^	0.198^∗^	0.225^∗^
Vitality (MSEI)	−0.067	0.025	0.104

*^∗^p < 0.05; ^∗∗^p < 0.01; ^∗∗∗^p < 0.001. DEBQ, Dutch Eating Behavior Questionnaire; INTE, Emotional Intelligence Questionnaire; IVE, Impulsiveness-Venturesomeness-Empathy Questionnaire; SPP 25, Resilience Measurement Scale; MSEI, Multidimensional Self-Esteem Inventory for general self-esteem, physical attractiveness, vitality.*

In women, significant but weak negative correlations were found between emotional eating and emotional intelligence and resilience. Higher impulsivity and general self-esteem in the women were associated with higher levels of emotional eating. A significant and moderate correlation was also shown between externalizing eating behaviors and impulsivity, where higher impulsivity was associated with higher levels of external eating. There was also a significant positive correlation between external eating and physical attractiveness, and a significant negative correlation between external eating and resilience. However, neither of these correlations were strong. There was also a significant positive correlation between general self-esteem and restrained eating in the women.

In men, significant and moderate correlations were shown between emotional eating and resilience and general self-esteem. Higher resilience was associated with lower emotional eating behaviors, but higher general self-esteem was associated with higher emotional eating behaviors. Significant positive but weak correlations were found between emotional eating attitude and impulsivity, and physical attractiveness. Significant but weak correlations were found between external eating and general self-esteem, and physical attractiveness. There was also a significant positive but weak correlation between general self-esteem including body image and restrained eating in the men.

However, the additional analysis, did not show significant differences between the correlations obtained in the women and men. Only the link between impulsivity and external eating differed between men and women; it was stronger in the women (*p* < 0.05).

### Psychological Predictors of Unhealthy Eating Attitudes in Young Women and Men

A structural equation analysis was performed to identify important psychological predictors of unhealthy eating attitudes, a path analysis was performed. Thus, the adopted theoretical model of independent variables (resilience, impulsivity), intervening (self-esteem, emotional intelligence) and dependent variables (restrained and external eating, emotional eating) was empirically verified (Figure 1). Results of the path analysis presented in Figure 1 and Table 7 allow the below assumptions to be adopted.

**Table 7:** Overall effects of the influence of variables (with due account of indirect and direct effect) represented in the path model.

Dependent variables	Impulsivity	Resilience	Emotional intelligence	General self-esteem
External eating	0.545	−0.059	in	in
Emotional eating	0.871	−0.235	in	0.685
Restrained eating	in	0.042	in	0.580

*in, insignificant.*

Impulsivity had the strongest and most direct significant influence on both emotional eating and external eating. In both cases the influence is positive, meaning higher impulsivity is associated with higher levels of emotional eating. Impulsivity proved not to have any significant impact on restrained eating. Resilience proved to have a significant impact on all eating attitude types, with a direct negative effect on emotional eating and external eating, meaning that higher resilience is associated with lower levels of emotional eating and decreased tendency to respond to triggers of smell, appearance and easy accessibility of food. Resilience also proved to have a low, yet significant positive direct effect on restrained eating, where greater resilience is associated with higher levels of restrained eating. Moreover, resilience proved to have a direct negative impact on self-esteem (including body self-esteem); and self-esteem as an intervening variable proved to have a strong positive effect on restrained eating. Higher general self-esteem and body self-esteem were associated with restrained eating. Interestingly, resilience proved to have a significant positive direct effect on emotional intelligence, yet emotional intelligence did not act as an intervening variable that would explain any of the examined types of eating attitudes. In turn, impulsivity also proved to have a significant direct yet negative effect on emotional intelligence, with emotional intelligence showing no significant correlation with eating attitudes. Only self-esteem proved to act as an intervening variable in compulsive eating attitudes. These results suggest that emotional intelligence has no evident impact on eating attitudes.

## Discussion

Men and women presented similar levels on all measures. Comparisons of correlation coefficients did not show relevant significant statistical differences by sex. Impulsivity is indeed more related to external eating in women. However, it is also significantly related to the second composition of compulsive eating attitude – emotional eating, both in women and men. This configuration of psychological variables allows one to describe the women and men in the sample as a homogeneous group of individuals who do not manifest eating disorders, which confirms accurate sampling for the study. The configuration of intensity for all measures confirms results often obtained by individuals in a healthy population (people who do not manifest various types of eating disorders), which also is reflected in research conducted by Boyd (2006), who showed that compared to individuals suffering from eating disorders, healthy people show less disordered emotional functioning, higher ability to cope with negative emotions in stressful situations, better ability to cope with stress and lower level of psychopathological traits. Other studies have also indicated that psychopathological traits of excessive anxiety, depression, impulsivity, emotional dysregulation, and negative coping are important criteria that differentiate healthy people from those with eating disorders (Grant et al., 2002; Jacobi et al., 2004; Boyd, 2006; Tchanturia et al., 2012; Torres et al., 2015; Gorwoord et al., 2016; Levinson and Rodebaugh, 2016; Brosof and Levinson, 2017; Wang and Li, 2017; Westwood et al., 2017; Brown et al., 2018).

There was a high level of resilience in our study participants, below-average general self-esteem combined with body self-esteem. This result may be specific to this particular sample of young educated individuals (mainly students). However, the results may also suggest that, regardless of their personality traits, the participants demonstrated lowered self-esteem when assessing themselves and their bodies (physical attractiveness) as well as poor acceptance of their own bodies. A more in-depth study and verification is required to identify whether the results indicate strong social-cultural influence on the assessment of body attractiveness for a young person.

The original study findings also indicated a significant positive correlation between resilience and emotional intelligence, which seems understandable and is also reflected in other research, showing that a high level of resilience is positively correlated with emotional intelligence, helping to cope with the experienced negative emotional states, ensuring hope and facilitating problem-solving in crisis situations (Curran et al., 2006). The original study findings also showed a significant correlation between participants’ impulsivity levels and their engagement in compulsive eating. Similar findings confirming that impulsivity regulates the release of experienced emotional states through compulsive (uncontrolled) binge eating (emotional eating) can be found in many studies (Macht, 2008; Ouwens et al., 2008; van Strien et al., 2013, 2012; Dingemans et al., 2017). Many studies also confirm the importance of emotional disorders in binge eating and bulimia (Pascual et al., 2011; Svaldi et al., 2012; Lavender et al., 2015). In turn, the correlation between impulsivity and external eating is also present in other studies (Rosval et al., 2006). It should be noted that impulsivity proved to have a significant (but not very strong) negative direct impact on emotional intelligence, in our original study. In modern literature, we can also find research that reports significant relationships between the manner of coping with stress and low emotional intelligence in people with eating disorders (Boyd, 2006; Matheson et al., 2012; Zysberg and Tell, 2013). We also encounter research reporting that in people with a strong restrained eating attitude, suppressing and denying their own emotional states and various stimuli coming from the body may result in lower emotional intelligence (Eizaguirre et al., 2004; Hambrook et al., 2012).

In previous literature we can find studies showing that low intelligence and negative affect combined with lack of capacity to regulate one’s own emotional states can significantly impact disordered eating patterns (Markey and Vander Wal, 2007; Koifman and Thomas, 2008; Matheson et al., 2012; Zysberg and Rubanov, 2010; Zysberg and Tell, 2013). Interestingly, the findings in the original study suggest that emotional intelligence proved not to have a significant direct effect on restrained and compulsive eating. However, it remains significantly correlated with impulsivity. Thus, there is a significant negative correlation: the higher impulsivity, the lower emotional intelligence – which does not have to stand for simultaneous existence of a tendency to resort to compulsive or restrained eating. Perhaps, this result shows that for exposition of compulsive or restrained eating emotional intelligence has a lesser impact than other variables in the original research model. Nonetheless, one should reflect on these findings in the context of slightly different results obtained by some other researchers. As was already mentioned above, in this original study impulsivity proved significantly correlated with emotional intelligence, yet not correlated with restrained and compulsive eating. This result does not exclude the possibility that impulsivity and emotional intelligence may affect other types of impulsive behaviors, such as aggressive and self-aggressive behaviors. Here, it is worth referencing studies that show impulsivity is correlated with a tendency to take excessive risk and an inability to postpone gratification (Moeller et al., 2001), as well as correlations with aggressive and self-aggressive behaviors (Jakubczyk and Wojnar, 2009). There is research that confirms the meaning of impulsivity in generating compulsive eating attitudes in people suffering from bulimia (Grzesiak et al., 2008; Manwaring et al., 2011; Forney et al., 2014; Lavender and Mitchell, 2015), as well in people who engage in binge eating (Izydorczyk, 2011, 2013). However, it should be recalled that the study group did not include participants with symptoms of eating disorders, which may be why study participants did not show a significant correlation between level of emotional intelligence and level of restrained and compulsive eating. One should also take into account research limitations stemming from the small number and specificity of the study group and assume that the above findings may be specific to that group. The findings show that aside from impulsivity, resilience acts as an important predictor in explaining both compulsive and restrained eating attitudes in young people (both men and women). The strength of influence of resilience on all three types of unhealthy eating attitudes is also confirmed by the finding that resilience had a significant indirect effect by means of self-esteem on restrained eating and emotional eating. In the case of the indicated correlations, resilience proved to be negatively correlated with external and emotional eating, meaning that the higher the resilience, the lower the intensity of the above-indicated unhealthy eating attitudes. If one adds to this the other result that showed greater resilience was associated with lower self-esteem, there appears to be a need to explain this state of affairs. On the one hand, we can say that this result may be characteristic only for the examined sample and may be the result of unintended methodological errors in how the research procedure was carried out. On the other hand, it is worth subjecting it to a more in-depth analysis with regard to psychological and socio-cultural functioning of the study participants, who were raised in a Western culture, where the binding body image standards serve as a significant indicator of general self-esteem (Izydorczyk and Sitnik-Warchulska, 2018). Socio-cultural impact on young adults promotes standards of success, ambition, and concentration on appearance and body. The below average self-esteem among the participants would confirm their tendency to lower their self-esteem, and would confirm that the obtained result indicated a tendency to prefer compulsive attitudes (Tables 2–4). According to the psychoanalytic perspective, compulsive eating can be used to produce a sense of feeling alive and build self-esteem (make the life sweeter) (Jacoby, 2017). Such results may indicate the narcissistic character of self-esteem in contemporary young adults. The relationship between self-esteem and narcissism as predictors of eating disorders was indicated by Boucher et al. (2015). Compulsive episodes of eating may, according to Tamhane (2017), be a continuation of restrictive eating behavior, helping to maintain self-esteem in the context of the need to implement sociocultural patterns of appearance. However, the findings warrant future research.

The significant positive correlation between self-esteem and emotional eating, and external, and weaker, but still significant, with restrained eating confirmed in the original study together with the above-listed arguments allows one to verify the meaning of socio-cultural influence on self-esteem, (including body image) and conclude that the value of this measure may remain negatively correlated with the obtained high score for resilience among study participants. The measurement of resilience levels may confirm high psychological resources of the examined young adults, whereas their resources pertaining to the capacity to evoke positive emotions, coping with stress, overcoming frustration, etc., do not necessarily need to act as a safeguard against the socio-cultural influence of body image standards on general self-esteem (which is very strong in that particular phase of life), strengthening lack of acceptance and not self-acceptance (according to a common standard of thinking ‘I am what I look like’). As shown by other studies, low self-esteem in young people, particularly women, which manifests in lower self-satisfaction, lower sense of self-worth and self-acceptance, constitutes a significant predictor for eating disorders (Stice, 2002). Low self-esteem is significantly correlated with disordered eating patterns such as anorexic attitude or binge eating (Paterson et al., 2007). Excessive self-criticism may lead to increased depressive states, overestimating one’s weight and a distorted view of one’s own body (Fairburn and Harrison, 2003), which may contribute to restrained eating attitudes. However, there are studies showing that mental resilience protects against self-assessment of the body (McGrath et al., 2009; Choate, 2011).

The authors of this study find it difficult to unequivocally interpret the results of their original study. Without doubt, the search for answers regarding the strength and direction of correlations between self-esteem and resilience requires further research and a more precise research model expanded to involve a more extensive and separate measurement of body image and self-esteem in both young women and men.

### Limitations, Implications, and Future Directions

The conducted studies were characterized by certain limitation that pertained to both sampling and the research procedure. First, the sampled study group (despite being sampled in line with the objective and the required research procedure) could constitute a specific group of women and men from a specific background, which could limit the interpretation of the results to other populations. However, the maintained common socio-demographic criteria for sampling and the number of participants support the reliability of the conducted study.

Second, the current study relied on self-report measures. Although clinical psychology assumes that one’s own perception is the most important for the direction of life, future research should use additional methods, such as behavioral assessment. Third, the study group was limited to one period of life, without disorders, and with a normal BMI. It would be interesting to compare the studied group with people in other developmental periods or presenting disordered eating behaviors (e.g., with the diagnosis of eating disorders or other mental disorders). Research on the dynamics of psychological processes and motivation for engaging in eating attitudes, particularly in the aspect of seeking their psychological predictors, would require future longitudinal studies, which are difficult to carry out. These would be more reliable and precise in assessing the research material. Nonetheless, the time-consuming character of such studies and the limited ability to conduct them, this form of research procedure was rejected. With the pre-set research objectives and the research procedure in mind, as well as with due account of methods for measuring variables acknowledged in the literature it was concluded that the adopted assumption and research procedure could be implemented by means of transversal studies.

However, it is worth pointing out that the presented research concerned a group of young adults, which does not happen often. A number of selected personal factors have been analyzed, which are indicated in the literature as important for health and eating behavior. A group of healthy people was examined, which may be a source of effective preventive methods, especially in the area of eating disorders. This seems particularly important in the context of the results related to a negative correlation between resilience and self-esteem and a positive correlation between self-esteem and compulsive and restrained eating attitudes. The idea of a relationship with the influence of socio-cultural and narcissistic self-assessment should be taken into account (see Discussion).

Future longitudinal studies on a study group of young men and women in long-term research relation (although difficult to implement) would ensure a more extensive measurement of processes underlying the development of unhealthy eating attitudes.

## Conclusion

To sum up the findings of our original study and the performed analyses, we can put forward the following conclusions.

First, resilience and impulsivity are psychological predictors that significantly and directly explain unhealthy eating attitudes, both limited food intake in daily diet (resilience) and compulsive eating (impulsivity and resilience). The higher the resilience, the higher the tendency to restrain eating, and lower to compulsive eating. High level of impulsivity are associated with high levels of compulsive eating.

An important psychological intervening variable in generating unhealthy eating attitudes proved to be a higher level of self-esteem among young people, both men and women. Emotional intelligence, which remains correlated with resilience, proved an independent variable with no effect on unhealthy eating attitudes.

Second, no significant differences were observed between the female and male participants regarding psychological variables they manifested, which were verified in the research model: resilience, self-esteem, and impulsivity, and regarding manifested restrained eating and emotional eating. Beyond one dependence, studied women and men did not differ in correlations between psychological factors and compulsive and restrained eating. The women’s and men’s emotional eating attitude positively correlated with self-esteem, and negatively correlated with resilience and impulsivity. Young women proved to have a higher dependence between impulsivity and a type of compulsive eating, that is external eating. This kind of eating attitude is related to body image in women and men. Body image also seems to be important for men using restrained eating behavior.

The study findings may support promotion of preventive treatment and educational programs implemented particularly among adolescents and young adults to support development of psychological resources (resilience, self-esteem). One should notice that the study findings are useful in raising awareness on the function of food in daily life (biological, emotional, and social) proposed in educational programs for adolescents and young adults to prevent growth of unhealthy eating patterns and development of eating disorders stimulated by socio-cultural factors. Preventive programs should include increasing resources such as resilience, as well as awareness of the presented self-esteem (including body image) in the context of socio-cultural standards that are currently promoted.

## Data Availability

The datasets generated for this study are available on request to the corresponding author.

## Author Contributions

BI contributed to the conception, design, and planning of the study, analysis of the data, interpretation of the results, drafting of the manuscript and revising it critically for important intellectual content, final approval of the version to be published, and agrees to be accountable for all aspects of the work. KS-W contributed to the conception and design of the study, interpretation of the results, drafting of the manuscript and revising it critically for important intellectual content, final approval of the version to be published, and agrees to be accountable for all aspects of the work. SL contributed to the conception and design of the study, analysis of the data, final approval of the version to be published, and agrees to be accountable for all aspects of the work. AL contributed to the conception of the study, acquisition of the data, final approval of the version to be published, and agrees to be accountable for all aspects of the work.

## Conflict of Interest Statement

The authors declare that the research was conducted in the absence of any commercial or financial relationships that could be construed as a potential conflict of interest.
